# Histological Tracking into the Third Dimension: Evolution of Early Tumorigenesis in VHL Kidney

**DOI:** 10.15586/jkcvhl.v8i3.189

**Published:** 2021-09-10

**Authors:** Mayyan Mubarak, Nayef Al-Gharaibeh, Samuel Sommaruga, Jie Li, Alexander Oliver Vortmeyer

**Affiliations:** 1 Department of Anesthesiology, King Faisal Specialist Hospital and Research Centre, Riyadh, Saudi Arabia;; 2 Department of Pathology, Indiana University Purdue University Indianapolis, IN, USA;; 3 Department of Physiology and Biochemistry, Jordan University of Science and Technology, Jordan;; 4 Department of Medicine, University of Geneva, Switzerland;; 5 Tianjin Key Laboratory of Clinical Multi-omics, Tianjin, China

**Keywords:** VHL, renal clear cell carcinoma, clear cell, carcinogenesis

## Abstract

Using a novel three-dimensional (3D) approach, we tracked histological changes to elucidate the earliest stages of renal clear cell neoplasia in normal kidney tissue of patients with von Hippel-Lindau (VHL) disease. Tissue blocks of interest were procured, serially sectioned, and 3D reconstruction of the entirety of pathologic events was performed. The results reveal an abundance of foci with aberrant clear cell proliferation that initially develop along the tubular lining, but have the potential to aggregate within individual tubules. This stage is followed by the extension of clear cell aggregates beyond the tubular basement membrane, which allows for the recruitment of angiogenesis derived from interstitial vasculature. The results suggest that the most frequent pathologic event in VHL kidneys is the presence of isolated or aggregated clear cells within the tubular epithelium, potentially developing further into a protracted process of neoplasia. The abundance of independent pathologic events in VHL kidneys confirms developmental mechanisms to precede tumor initiation. To our knowledge, this is the first report demonstrating that tracking of histologic changes in the 3rd dimension enables the confirmation of the sequence of events from the earliest pathologic change in the VHL kidney to the neoplastic stage. This approach is not only useful for visualization and quantification of pathologic changes but also for targeted sampling allowing selective analysis of the earliest stages of clear cell carcinogenesis.

## Introduction

Patients with VHL disease develop tumors in the nervous system, kidneys, endolymphatic sac, epididymis or broad ligament, neuroendocrine tumors, and pheochromocytomas (1). The presence of abundant microscopic tumor precursor structures in the nervous system of patients with von Hippel-Lindau (VHL) disease has been previously demonstrated (2, 3). The cells of these precursor structures were shown to be immature (3, 4), and several lines of evidence suggest that pathologic cells were generated during early organ development (5). The growth pattern of precursor structures appears highly protracted, with only a subset of them developing into frank tumors (3). Precursor structures defy categorization as teratomatous, tumorous, or dysplastic, and the designation of a developmentally arrested structural element (DASE) has also been previously suggested (4). In VHL disease, the inactivation of the VHL wild-type allele (“second hit”) is a prerequisite for the formation of DASEs. Since a majority of the DASEs never develop into frank tumors, two-hit inactivation of the VHL gene is necessary but insufficient for tumor development. A key question remains the nature of the “third hit” that would transform DASEs into frank tumors.

Similarly, the epididymides of male VHL patients reveal VHL-deficient, developmentally aberrant structures that may give rise to frank tumors (6). In VHL disease, both the epididymides and the kidneys independently generate mesonephros or metanephros-derived tumors (1). Interestingly, epididymal neoplasia is generally benign, whereas renal clear cell carcinoma is associated with metastatic spread, and significant morbidity and mortality.

Seminal studies that have demonstrated abundant foci of clear cell proliferation in the VHL kidney (7, 8) had prompted us to investigate on a better characterization of these proliferations and determine the sequence of their evolution. We started investigating tissue sections from tumor-free, normal-appearing kidney tissue for developmental aberration and earliest steps of tumorigenesis (unpublished data), in analogy to previous studies on other tumorigenic sites in VHL disease, for better clarification of the earliest stages of renal tumorigenesis in VHL disease (1). Most frequently observed pathologic events in VHL kidney sections were either isolated clear cells within a renal tubule or two or more clear cells that were adherent to each other, likely representing the result of an amplification event. Higher accumulations of clear cells along the tubular lining and clustering of clear cells within renal tubules appeared to be in the continuity of smaller preceding events.

Previous studies, however, performed an analysis of single random tissue sections that only allowed for limited interpretation of the evolution of histologic changes (7, 8). Using a novel three-dimensional (3D) algorithm consisting of methodological sampling and histological tracking (later developed in the text), we tracked those lesions to confirm their relationship to each other. This algorithm allowed us to observe that microscopic clear cell proliferation followed a predictable pattern covering an entire morphological spectrum of histologic lesions from an isolated clear cell to tumorigenic clear cell proliferation.

In this study we first verified the reproducibility of our 3D algorithm developed on one VHL kidney (training set) on a larger cohort of VHL and non-VHL kidneys (validation set) from the inspiration of the presentation of the results at the 2014 VHL meeting in Madrid (9) along with a review of hypothetical histological pathways (10).

## Materials and Methods

### Tissues

Seven kidneys of seven different patients were obtained at autopsy and used for this study after complete fixation in 10% buffered formalin. Four kidneys were from patients with germline mutation of the VHL gene and established diagnosis of VHL disease (designated as “VHL kidneys”). All patients had different VHL disease-associated tumors including renal clear cell carcinoma (CCC), and had died between ages 50 and 65 years. Three control kidneys from patients with sporadic renal cell carcinoma, aged between 55 to 70 years, were also used for this study (designated as “sporadic kidneys”).

The kidneys used for this study had already been partially sectioned for original diagnostic evaluation, and portions of tumor nodules had been excised for histologic confirmation of renal CCC. In general, VHL kidneys showed involvement by numerous tumor nodules and cysts of variably size. In contrast, sporadic kidneys showed a solitary cancerous nodule, whereas the remaining kidney tissue appeared without pathologic changes.

### Processing of kidneys

The primary goal of this study was to identify and analyze renal tissues with no or only minimal grossly visible pathologic changes. All kidneys were sectioned randomly into maximally 10 cm × 1.5 cm × 1.0 cm columnar cuboids. The obtained cuboids of each kidney were inspected, and cuboids showing any grossly visible tumor were rejected. Of the remaining cuboids, the cuboid with the least grossly visible pathologic changes was identified and selected as the cuboid of interest (COI). For each kidney under study, one COI was selected (n = 7).

COIs were then sliced into 0.3 cm × 1.5 cm × 1.0 cm blocks and a maximum of 30 blocks per COI was created. All tissue blocks without grossly visible pathologic change were paraffin-embedded. An initial section was taken from each block and stained with hematoxylin and eosin (H&E) for histologic evaluation and identification of the block of interest for 3D reconstruction.

### Block of interest (BOI)

The purpose of the BOI selection was to identify the most informative kidney tissue block for 3D reconstruction for each case under study. The initial H&E sections for each block allowed for examination of the extent of unspecific pathologic changes including reactive inflammation, fibrosis, and/or hemorrhage. Tissue blocks of some COIs were rejected because of the presence of unspecific pathologic changes. Among the acceptable tissue blocks, blocks containing renal cortex were selected, and a BOI was selected based on the absence of unspecific histopathologic changes and the quality of histological preservation. For each case, one BOI was subjected to 3D analysis.

### 3D reconstruction of pathologic events

Each BOI was serially sectioned into 5 um-thick sections, and each section was placed on an individual glass slide and labeled consecutively. Every tenth section was stained with H&E and scanned using a Ventana iScan slide scanner (Roche, Basel, Switzerland). Unstained sections were preserved for possible follow-up studies.

All H&E slides were consecutively evaluated for cytologic and structural differences, compared with regular renal histology using high power magnification. Any cytologic/structural difference was separately annotated and categorized using iScan image viewer software. This process allowed us to obtain a pathologic event count within the BOI, which could be compared with sporadic or other VHL kidneys (see below).

### Quantitative assessment of pathologic events

The most frequently observed pathologic events in the VHL kidney sections could be defined either as an isolated clear cell within a renal tubule or as two or more clear cells adherent to each other, likely representing the result of an amplification event. Since the microscopic clear cell proliferations followed a predictable pattern, all pathologic clear cell events could be conveniently separated in five types of pathologic lesions by considering the lesion presentation and size in ascending order: (i) Type A-single clear cells; Type B-small chain of clear cells; (iii) Type C-large chain of clear cells; (iv) Type D-intratubular clear cell cluster; and (v) Type E-invasive clear cell cluster.

All type C to E events were independently annotated and counted in each consecutive section. If a deeper section showed the continuation of an event, the event retained the same annotation. By this approach, we were able to count the exact number of events in each BOI. Type A and type B events were so abundant that 3D counts were obtained from a 1mm × 1mm representative area within the section and from the corresponding 1mm x 1mm areas in the consecutive sections. In other words, a 1mm × 1mm × 3 mm (BOI thickness) column designated as “micro-BOI” was assessed within the BOI. For type C lesions, both BOI and micro-BOI counts were obtained to allow separate comparison with either type A and B or type D and E events. Microsoft Excel software was used for the documentation and graphics.

## Results

**Figure 1:**
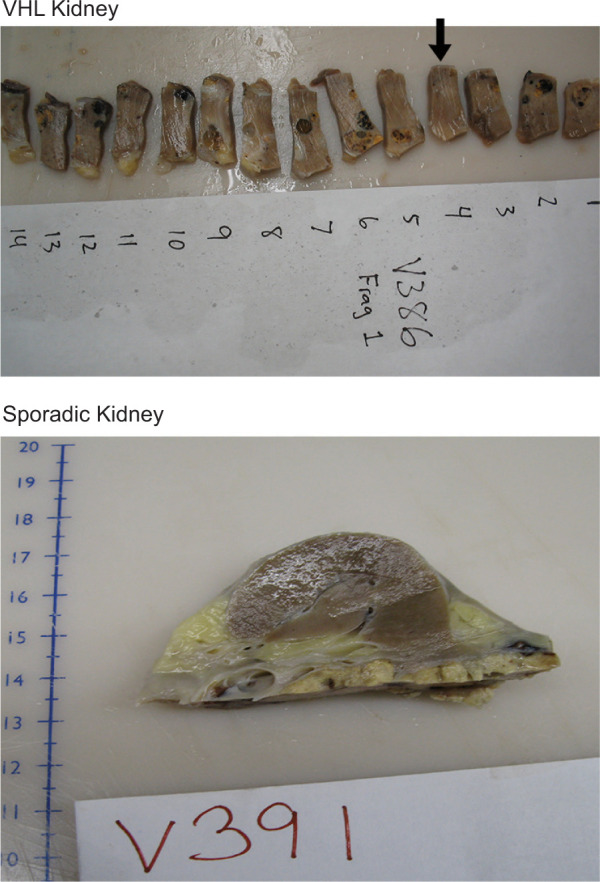
Example of VHL kidney cuboid, sectioned into consecutive tissue blocks (top); cystic structures or small tumor nodules are visible in most of the blocks; chosen tissue blocks of interest (BOI, arrow) show no or only minimal pathologic changes grossly; in contrast, sporadic kidney reveals no obvious pathologic changes in non-tumorous parenchyma (bottom).

**Figure 2:**
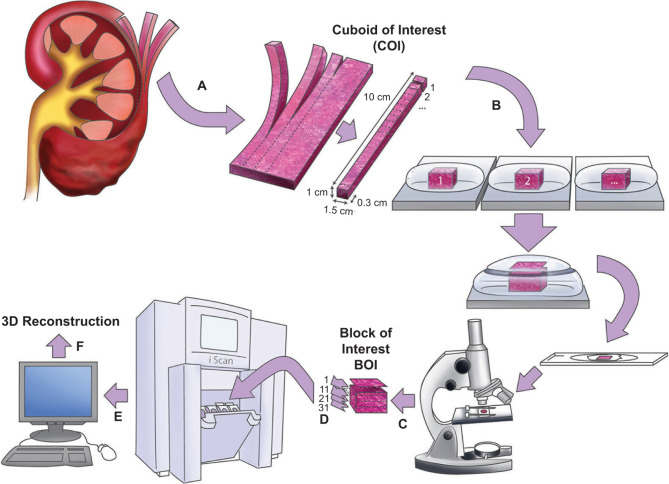
Processing of sporadic and VHL kidneys; A, slicing of kidney into cuboids is followed by selection of cuboid of interest (COI); B, sectioning into tissue blocks is followed by selection of normal-appearing blocks and creation of initial HE-stained section for selection of block of interest (BOI); C, serial sectioning of BOI and staining of representative sections; D, scanning of representative sections; E, evaluation and annotation of HE-stained sections; F, 3d tracking and reconstruction of pathologic events along consecutive levels.

**Figure 3:**
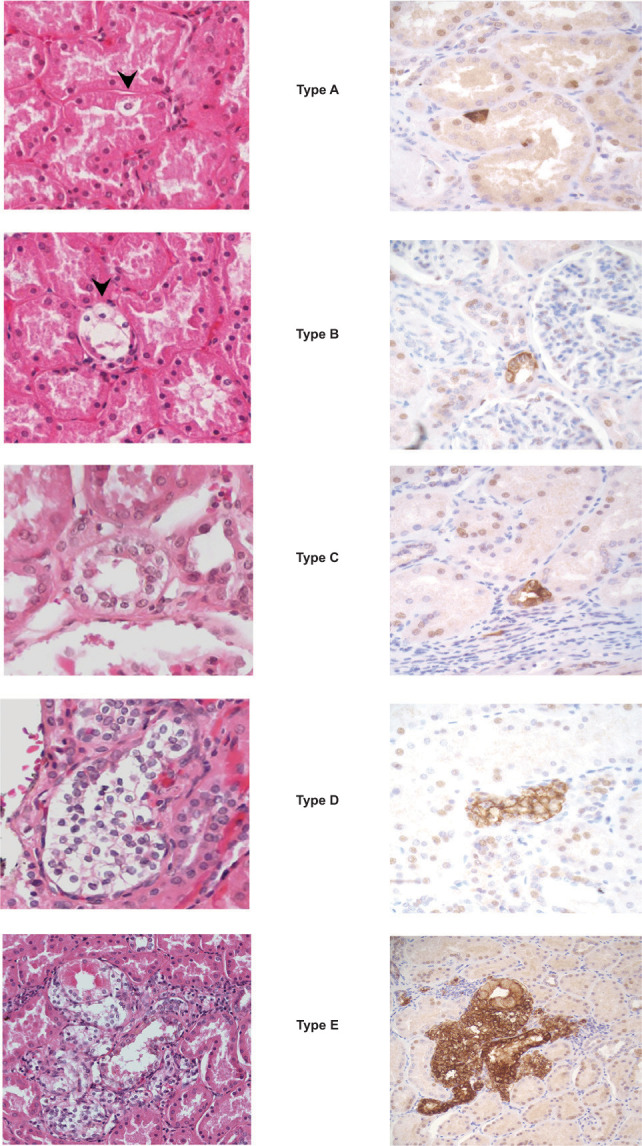
Most frequently observed pathologic events in VHL kidney. Events can be identified on H&E-stained sections (left) or by immunohistochemistry for CAIX (right). Representative samples of sections unrelated to each other are shown. Type A, single clear cell; type B, small chain of clear cells; type C, large chain of clear cells; type D, intratubular clear cell cluster; type E, invasive clear cell cluster.

The gross appearance of VHL kidneys was different from that of sporadic kidneys. VHL kidneys contained numerous diffusely scattered small tumor nodules, whereas sporadic kidney tissue showed a solitary tumor only (Figure 1). As previously processed with our training set, kidneys were sliced, and the slices were subdivided into cuboids. The selected cuboids of interest were subdivided into tissue blocks from which tissue BOIs were identified and subjected to serial sectioning and 3D analysis (Figure 2). Upon histologic investigation, an abundance of microscopic pathologic events was identified and annotated. The majority of the pathologic events were characterized as variable amounts of intratubular clear cells. Figure 3 shows the categorization of the event into five different types, reflecting ascending order in sizes.

### Type A: an isolated clear cell within a renal tubule

**Figure 4:**
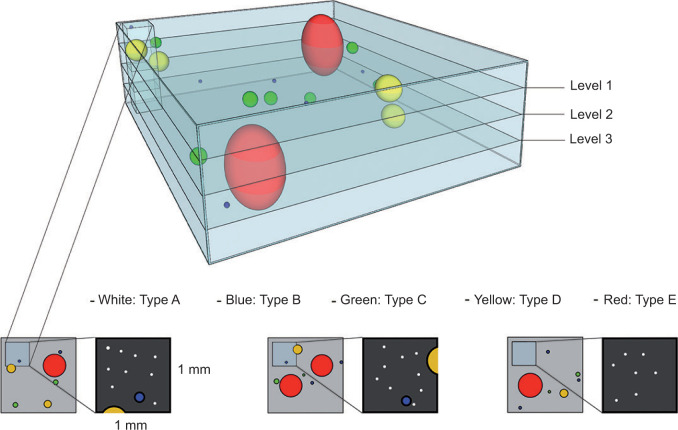
Diagram of VHL kidney block of interest containing pathologic events of different sizes which are displayed in different colors. The histologic presentation of different section levels into the block is shown (annotated as “Level 1”, “Level 2”, “Level 3”). Also demonstrated is the micro-BOI column which was represented by a 1mm x 1mm field on each consecutive slide and used for counting type A, B and C events. Consecutive sections through the block allowed for reconstruction of size and shape of each annotated pathologic event.

**Figure 5:**
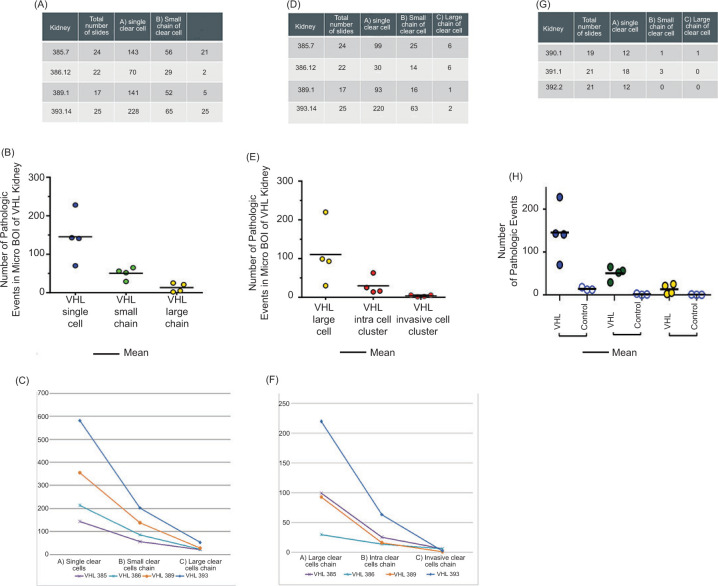
Numbers of pathologic events in micro-BOIs (left, A-C) and BOIs (middle, D-F) in VHL kidneys and control kidneys. Control results are only shown for micro-BOI counts (right, G, H); control results were consistently negative for large chains, intratubular clear cell clusters or invasive clusters. In all blocks, lower complexity events numerically exceed consecutive events of higher complexity.

Clear cells are a well-known phenomenon in the VHL kidney (8). They have been identified as VHL-deficient, with activation of hypoxia-inducible factor (HIF) and upregulation of target proteins including carbonic anhydrase IX (CAIX) (8). An empty appearance and enlarged cytoplasm with a round nucleus denote a clear cell (Figure 3). Among the annotated pathologic events, single clear cells (type A events) were the most frequent. The number of single clear cells was counted within a micro-BOI representing 1% of the block volume (Figure 4). Within the evaluated micro-BOI areas, the numbers of isolated clear cells ranged from 70 to 228 in four VHL kidneys (Figure 5). In contrast, only six to 12 isolated clear cells were identified in sporadic kidneys.

### Type B: small clear cell chain

A small clear cell chain is two or more clear cells that are adherent and replace less than 50% of a tubular circumference (type B event, Figure 3). Like type A events, they were also counted within the micro-BOI column. Within the selected micro-BOIs of VHL kidneys, the numbers of small clear cell chains ranged from 29 to 65 events. In contrast, the micro-BOIs of sporadic kidneys contained no or maximally one event (Figure 4).

### Type C: large clear cell chain

A large clear cell chain has multiple adherent clear cells that replace more than 50% of the tubular circumference (Figure 3). Type 3 events were separately counted in both the micro-BOI column and in the entire block. Within the selected micro-BOIs of VHL kidneys versus the entire section surface, the numbers of large clear cell chains ranged from two to 25 events versus 99 to 220 events (Figure 4). In contrast, the micro-BOIs of sporadic kidneys contained no or maximally one large clear cell chain event versus none.

### Type D: intratubular clear cell cluster

Intratubular clear cell clusters were defined as clusters of clear cells that filled and frequently expanded a renal tubule (Figure 3). In contrast to type B and C events, the intratubular clear cell cluster showed multilayered growth from two layers to full lumen occupancy and occasional tubular expansion. The entire surface of each section was evaluated. Between 14 and 63 intratubular clear cell clusters were detected in the BOIs of VHL kidneys, whereas none were found in control sporadic kidney (Figure 4).

### Type E: invasive clear cell cluster

Intratubular clear cell clusters (Type D) were frequently observed in VHL kidneys. Upon analysis in 3D tracking, we identified occasional and focal destruction of tubular epithelium and basement membrane indicative of amplification, proliferation, and invasion of clear cells beyond the basal membrane into the renal interstitium separately categorized as Type E, invasive clear cell clusters (Figure 3). Between 1 and 6 Type E events were detected in four VHL kidneys. Type E events were absent in non-VHL control kidneys.

## Discussion

Renal tubular clear cell change resembling VHL-deficient clear cells can occur secondary to metabolic/toxic etiologies. These include changes secondary to toxins and Alport nephropathy (11–13), osmotic tubulopathy seen in diabetes and acute kidney injury (13) or changes after infusion of radiologic contrast media (14). These changes show prominent cytoplasmic vacuolization with preservation of the epithelial brush border. In contrast, VHL-deficient clear cells are without brush border or any other morphologic evidence of tubular differentiation. They are distinct from tubular epithelium, which appears to be gradually replaced by proliferating clear cells. Further, VHL-deficient clear cells show consistent strong positive immunoreactivity with anti-CAIX (8). This consistent immunoreactivity with anti-CAIX differentiates VHL-deficient clear cell clusters from clear cell clusters in the sporadic kidney, which occur rarely and are negative for CAIX (15). We did not detect any clear cell clusters in our three sporadic, serially sectioned control kidneys.

Application of imaging techniques established early on that renal tumors in VHL disease frequently occur in multiplicity (16). Most radiologically detected lesions are small and can be surveilled until the index tumor reaches 3 cm in size, at which malignant metastatic spread can occur (17). On purely histopathological criteria, precancerous and precursor lesions in the VHL kidney have been previously appreciated (18, 19).

Beyond analysis of grossly or radiologically visible tumor analysis, Walther et al. (7) investigated normal-appearing renal tissues from VHL patients (7). In the “grossly normal” VHL kidney, investigators detected an abundance of small clear cell tumors in the VHL kidney with partial resemblance to invasive clear cell clusters identified in this study. Single H&E-stained sections of paraffin blocks were investigated to document and extrapolate the frequency of tumors and were estimated as 600 per VHL kidney (7).

In selected sections of VHL kidney, Mandriota et al. (8) described an abundance of either isolated or small chains of tubular cells with VHL gene inactivation. Clear cells were confirmed to be positive for CAIX as a consequence of VHL gene inactivation. By laser confocal microscopy and immunohistochemistry combined with fluo-rescent *in situ* hybridization, single CAIX-positive clear cells were subsequently confirmed to be VHL gene-deleted (20). Based on these and additional unpublished findings (9, 21), a developmental model has been suggested, proposing the progression of single VHL-deleted cells via cellular amplifi-cation to cysts and tumorlets (10).

By using 3D tracking, we demonstrate that early stages of cellular amplification occur along the tubular lining. A subsequent key stage appears to be a neoplastic progression into intratubular clusters, allowing for voluminous expansion. The genetic and epigenetic mechanisms of this event remain to be clarified. It is also unknown whether penetration of the tubular basement membrane occurs by an active invasion of transformed cells or results “passively” from uncontrolled expansion. Of particular interest in VHL disease has been the relationship between cysts and tumors. Further studies need to clarify whether cysts and tumors develop via independent mechanisms or are merely variations of an essentially uniform pathogenetic process. While consequences of dysregulation of VHL and HIF pathways provide numerous potential clues (10), our approach may be helpful to obtain *in situ* evidence in human tissues in the future.

**Figure 6:**
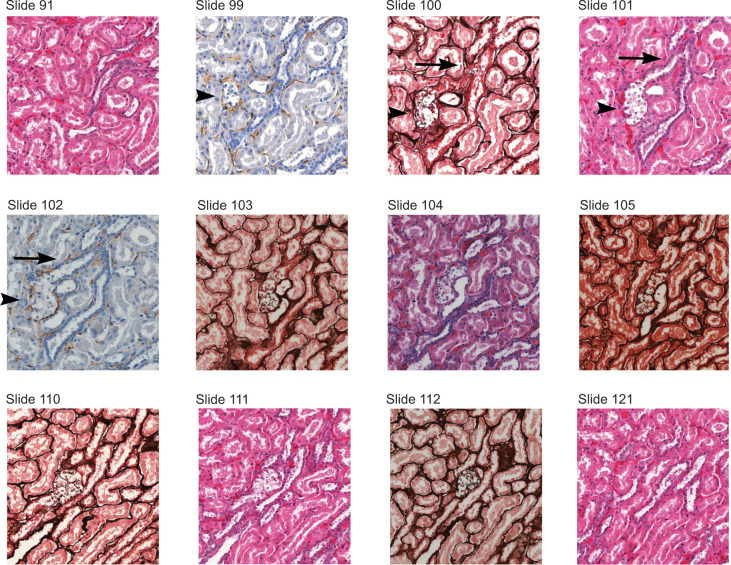
Selected images of 3D reconstruction of a single pathologic event in VHL kidney. The event was originally identified on slides #101 and #111 and classified as type D event (clear cell cluster). Consecutive unstained sections were used and variably stained for more detailed reconstruction. The event is contained between sections #91 and #121 which show no related pathologic change. The event is characterized by a tubule obstructed by clear cells (demonstrated in sections #99 to #112). While the tubule is expanded by the clear cell proliferation, Jones special stain reveals intact tubular basement membrane (slides #100, #103, #105, #110, #112). Immunohistochemistry for CD31 shows increased capillarization in direct vicinity to the clear cell cluster (slides #99 and #102) as previously shown by Mandriota et al.

Our 3D tracking approach allows us to significantly expand our understanding of early tumorigenesis by providing numerical quantification of pathologic events. By this quantification, we conclude that pathologic events of lower complexity are consistently more numerous than pathologic events of higher complexity. Structural details will allow for lesion volume calculation and observations of relationships between lesion volume and histopathological change. In addition, enhanced structural detail can be obtained by the utilization of adjacent sections that were initially kept unstained, but could be subjected to special stains or immunohistochemical preparations of interest later in time (Figure 6).

Our results demonstrate that smaller clear cell aggregates that precede tumorlet formation are numerically far more abundant. A prerequisite for our study was a procurement algorithm that provided the most informative tissues by targeted gross tissue examination and triage, followed by further quality triage by histological examination of selected blocks. In contrast to routine diagnostic tissue procurement, we triaged all tissue blocks to identify the most unremarkable-appearing and best-preserved representative tissue blocks for each kidney. The entire kidneys were sectioned into cuboids of 1.0 cm × 1.5 cm base area with a maximum of 10 cm in length. All cuboids containing visible pathology were rejected, and the most normal-appearing cuboid was selected from each kidney. Consecutive processing and microscopic analysis of all blocks sectioned from the selected cuboid allowed for the selection of BOIs for a more detailed study.

In the BOIs of VHL kidneys, we detected a frequency of smallest microscopic pathologic events (type A, B, C) that by far exceeded the frequency of clear cell tumorlet clusters, previously described by Walther et al. (7). More specifically, in all examined tissue blocks, the number of smaller-sized clear cell events consistently exceeded that of larger-sized events, providing evidence that clear cell structures develop from the amplification of single VHL-deleted clear cells. That only a tiny proportion of smaller-sized events progresses into larger-sized events provides further evidence for the protracted character of tumorigenesis, previously described in the nervous system of VHL patients (22). This protracted progression may be the morphological equivalent of a linear evolutionary subtype of tumor progression identified by whole-genome analysis of renal cancer tissues in VHL disease (23).

Our 3D tracking approach allowed us to visualize and examine many microscopic clear cell changes, which consistently revealed strict dichotomic presentation of VHL-deficient clear cells and normal tubular cells, without intermediary cytology or microarchitecture that would suggest transition of the renal tubular cell to clear cell. This outcome agreed to the microscopic change demonstrated by a previous study (8). While the number of invasive clear cell clusters in VHL kidney has been previously extrapolated as 600 approximately (7), the number of smaller clear cell events is exponentially larger. It thus remains challenging to explain how thousands or millions of tubular cells would independently be subjected to a second hit. A more compelling explanation for the frequency of VHL gene function loss is the occurrence of deletion events during metanephric renal development, resulting in persistent VHL-inactivated or stem cells within the tubular lining.

Evidence of different developmental states of proximal tubular cells has been provided, as putative progenitor cells have been identified in the human kidney, scattered throughout the proximal tubules (24, 25). In histological resemblance, the VHL kidney appears to represent a “mosaic” composed of wild-type and VHL-inactivated cells, with a small subset of VHL-inactivated cells amplifying along a defined sequence of progression.

Male patients with VHL disease develop CCC in the kidney and cystadenoma in the epididymis (6). Both tumors share a mesonephric origin but show distinct biological behavior with only CCC harboring malignancy potential. Of special interest may be the profound difference between the earliest neoplastic proliferation. Via hypoxic signaling beyond the basement membrane, early intratubular renal clear cell change is associated with angiogenesis in the adjacent stroma (8), without affecting tubular microarchitecture. In contrast, hypoxic signaling of early intraductal clear cell change in the epididymis is associated with micropapillary intraductal growth. The characteristic changes of mesonephric clear cell proliferation in the VHL kidney – replacement of tubular cells and clustering – are not present in the VHL epididymis. Of interest are, therefore, not only differences in proliferative behavior of VHL-deficient mesonephric cells but also differences in stromal response.

In summary, our approach to tissue grossing and 3D histological tracking aids in the detailed analysis of earliest cytologic and structural changes in organs affected by VHL disease. It allows for the identification of large numbers of potential precursor structures including numerical and structural quantification. In addition, histologically controlled unstained sections can be retained for additional studies including specialized histology or macro/microdissection for advanced analysis. Even though the applied approach is time-consuming, it has been highly rewarding. This study proved that the 3D algorithm used is reproducible, and the study findings are robust and reliable. We hope that this 3D algorithm will pave the road to better-targeted sampling for future studies, which will provide enhanced insights into VHL kidney tumorigenesis.
